# Association between perioperative zinc supplementation and recovery of walking ability after hip fracture surgery: a propensity score-matched study

**DOI:** 10.1007/s00264-026-06865-5

**Published:** 2026-05-26

**Authors:** Hironori Unno, Takahiro Hasegawa, Masayoshi Sato, Gai Kobayashi, Shine Tone, Yohei Naito, Masahiro Hasegawa

**Affiliations:** 1 Department of Orthopaedic Surgery, Mie University Graduate School of Medicine, Tsu City, Japan; 2 Department of Orthopaedic Surgery, Iga City General Hospital, Iga City, Japan

**Keywords:** Zinc, Hip fracture, Walking ability, Sarcopenia

## Abstract

**Purpose:**

Hip fractures are frequently associated with poor functional outcomes, partly due to underlying nutritional deficiencies such as zinc deficiency. However, the role of zinc supplementation in the postoperative functional recovery of patients with hip fractures remains unclear.

**Methods:**

This prospective observational study included 75 patients with hip fractures who were ambulatory before injury and underwent surgical treatment. The patients were allocated into a zinc supplementation group (group Z) and control group (group C). Group Z received oral zinc supplementation (50 mg/day) from admission until two weeks postoperatively. The primary outcome was the recovery of walking ability at discharge, defined as achieving a level equal to or better than the pre-injury status. The secondary outcomes included changes in walking score (Δ walking score) and serum zinc levels. Propensity score matching was performed to reduce confounding factors, resulting in 26 matched pairs. Multivariable logistic regression analysis was performed to identify the independent predictors of functional recovery.

**Results:**

Hypozincaemia was found in 98.7% of the patients at admission. After propensity score matching, the rate of walking ability recovery was significantly higher in group Z than in group C (61.5% vs. 26.9%, *p* < 0.05). The Δ walking score was also significantly greater in group Z. The change in serum Zn level from admission to two weeks postoperatively was significantly higher in group Z. Multivariable logistic regression analysis identified zinc supplementation as the only independent predictor of walking ability recovery (odds ratio, 3.74; 95% confidence interval, 1.03–13.70; *p* < 0.05).

**Conclusions:**

Perioperative zinc supplementation was significantly associated with improved walking ability recovery after hip fracture surgery. Given its simplicity and safety, zinc supplementation might be a practical strategy for improving postoperative functional outcomes in this patient population. Further large-scale randomized controlled trials are warranted to confirm these findings.

## Introduction

Hip fractures are a major global public health issue, often resulting in hospitalization, loss of independence, and death [1]. Notably, on admission, patients with hip fractures commonly present with geriatric nutritional disorders, particularly malnutrition and sarcopaenia. The presence of these disorders is strongly associated with poor surgical outcomes, increased complications, prolonged rehabilitation, and higher mortality rates [2]. Since many patients have significant nutritional deficits before surgery, improving their nutritional status solely through in-hospital nutritional interventions is challenging.

Zinc is a nutritionally essential trace mineral required for the function of more than 300 enzymes involved in major metabolic pathways. It is distributed throughout the body, with 85% located in muscles and bones [3]. Zinc deficiency causes a variety of symptoms, including skin and taste disorders, gonadal dysfunction in men, loss of appetite, diarrhea, delayed wound healing, and increased susceptibility to infection [4]. Moreover, individuals with sarcopenia exhibit significantly lower serum zinc levels compared with those without the condition [5]. Previous clinical studies in non-orthopaedic settings have demonstrated that zinc supplementation can promote wound healing, enhance immune function, and improve physical performance in older adults.

However, despite its biological plausibility and emerging clinical evidence from other medical fields, the potential role of zinc supplementation in patients with hip fractures has not yet been clarified. To the best of our knowledge, no previous study has specifically evaluated whether zinc supplementation could improve postoperative functional recovery in this patient population. Therefore, we hypothesized that zinc supplementation in patients with hip fractures would be associated with better recovery of walking ability after surgery.

## Material and methods

### Study design and patients

This prospective observational study included patients with hip fractures who were surgically treated in our department between December 2024 and November 2025. During the study period, 104 consecutive patients underwent hip-fracture surgery. Of these, 75 patients (mean age, 85.7 ± 7.1 years; 12 male and 63 female individuals) who were ambulatory before injury were eligible for inclusion in the study. Patients who were unable to walk before the injury were excluded.

### Fracture classification and surgical treatment

Femoral neck fractures were classified according to the Garden classification system.

Internal fixation with cannulated cancellous screws was performed for Garden type I and II fractures, whereas hemiarthroplasty was performed for displaced fractures classified as Garden type III or IV. The trochanteric fractures were surgically managed by proximal femoral nailing.

### Postoperative rehabilitation protocol

Postoperative rehabilitation was initiated as early as possible and continued until discharge. Patients were permitted full weight-bearing on the first postoperative day. During hospitalization, all patients underwent postoperative rehabilitation within the same institution according to a standardized clinical pathway. No patients were transferred to rehabilitation hospitals during the acute postoperative phase. The rehabilitation progressed in a stepwise manner, beginning with wheelchair mobility, followed by ambulation using parallel bars, a walker, a cane, and ultimately independent walking. The patients were discharged home or transferred to another facility for ongoing rehabilitation when required.

### Zinc supplementation and group allocation

The patients were divided into two groups based on their zinc supplementation status. The zinc supplementation group (group Z, n = 32) received one tablet of 313.4 mg zinc histidine hydrate contain 50 mg zinc from the day of admission until two weeks postoperatively. The control group (group C, n = 43) did not receive any zinc-related medications.

Serum zinc levels were measured on admission and also on awakening the day postoperative, one week and two weeks after surgery. The cutoff for hypozincaemia was defined as 80 ㎍/dL; the prevalence of hypozincaemia at the time of admission was investigated.

### Outcome measures

Walking ability was classified into six categories based on the level of walking assistance required: (1) Unassisted ambulation, (2) ambulation with a cane, (3) ambulation with a walker, (4) ambulation with a rollator, (5) dependence on a wheelchair, and (6) confinement to bed.

Improvement in walking ability was defined as the attainment of an equivalent or superior ambulatory category at hospital discharge, relative to the pre-injury baseline.

Regarding secondary outcome analyses, we quantified the changes in walking ability by a numerical walking score, calculated as the postoperative score minus the pre-fracture score and analyzed this as an ordinal variable. The walking score ranged between 1 and 6, with higher values reflecting greater independence (6 = unassisted walking, 5 = cane-assisted walking, 4 = walker-assisted walking, 3 = rollator-assisted walking, 2 = wheelchair use, and 1 = bedridden walking). Consequently, the change in walking score (Δ score) spanned from − 5 to + 5, where positive values indicated better functional recovery after surgery.

### Statistical analysis

Between-group comparisons were performed for age, sex, body mass index (BMI), fracture type (femoral neck or trochanteric), pre-fracture walking ability, length of hospital stay, serum zinc levels (on admission, the day after surgery, one week and two weeks postoperatively), change in serum zinc levels (difference between two weeks after surgery and the time of admission), improvement in walking ability, Δ walking score, and presence of perioperative complications.

Categorical variables were compared using Fisher’s exact test; continuous variables were compared using the Mann–Whitney U test. Multivariable logistic regression analysis was performed to identify independent factors associated with improvement in walking ability (binary outcome: improved vs. not improved). The model included age, sex, BMI, fracture type, presence of postoperative complications, and zinc supplementation status as covariates. The results are presented as odds ratios (ORs) with 95% confidence intervals (CIs).

To reduce potential confounding factors, propensity score matching was performed using age, sex, BMI, fracture type, and pre-injury ambulation status. A one-to-one nearest-neighbor matching without replacement was applied. All statistical analyses were performed using EZR software, and a *p*-value of < 0.05 was considered statistically significant.

### Ethical approval

This study was approved by the Institutional Review Board of the Human Investigation Committee of the Iga City General Hospital (code:2024–747, date: December, 18th, 2024).

## Results

The mean serum zinc level was 53.6 ± 12.0㎍/dL; hypozincaemia was found in 74 of 75 patients (98.7%).

After propensity score matching, 26 patients were included in each group. The mean age was 85.8 ± 6.1 years in group Z (4 male and 22 female patients) and 85.8 ± 5.7 years in group C (3 male and 23 female individuals), without statistically significant difference between the groups. There were no significant differences in BMI, fracture type, pre-fracture walking ability, or length of hospital stay between the two groups (Table 1).

**Table 1:** Baseline characteristics of the study population after propensity score matching

Parameter	Group Z (*n* = 26)	Group C (*n* = 26)	*P*-values
Age	85.8 ± 6.1	85.8 ± 5.7	0.83
Sex, n (male/female)	4/22	3/23	1.00
Body mass index (kg/m^2^)	19.7 ± 3.4	20.3 ± 3.2	0.40
Fracture type, n (femoral neck/trochanteric)	16/10	17/9	1.00
Pre-fracture walking ability	5.1 ± 1.1	5.1 ± 1.1	0.83
Length of hospital stay (days)	40.2 ± 15.1	42.0 ± 24.1	0.80

Data are presented as mean ± standard deviation or number. Fisher’s exact test and Mann–Whitney U test were used for comparison

**Fig. 1:**
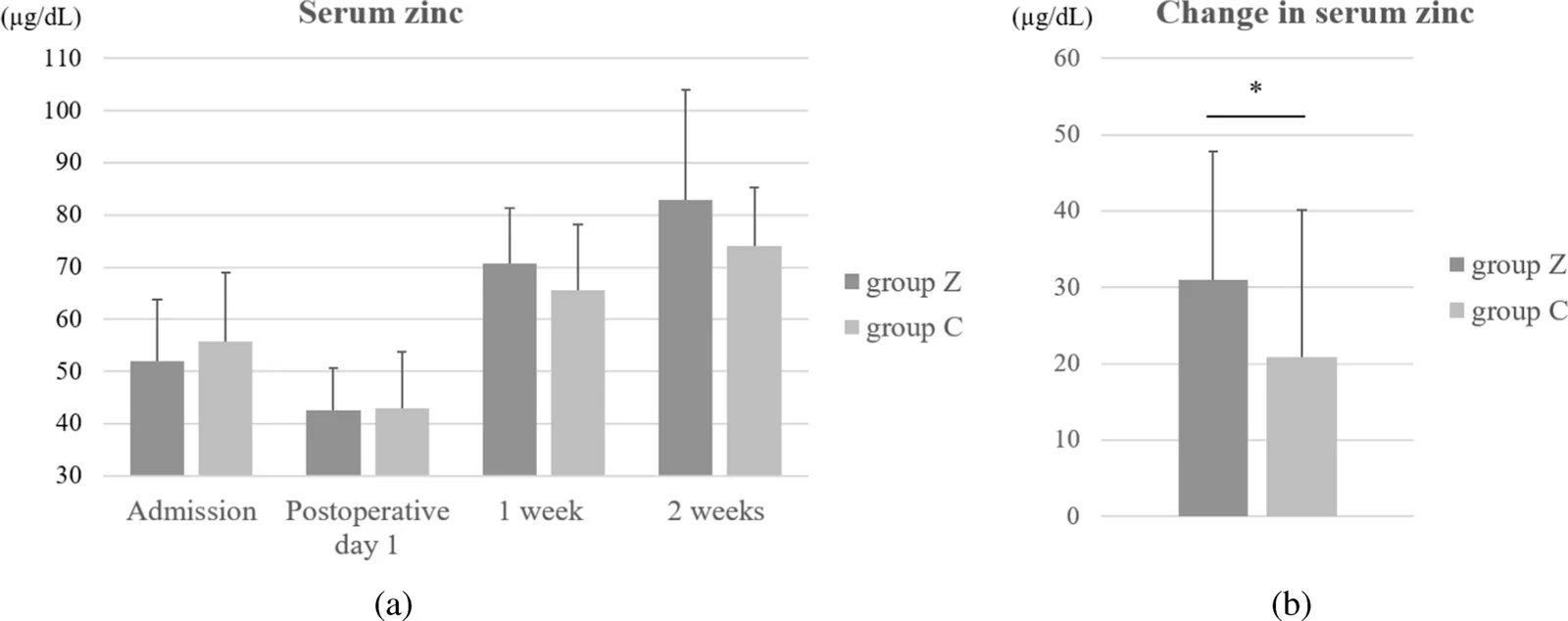
Changes in serum zinc levels. (**a**) Time course of serum zinc levels from admission to 2 weeks postoperatively in the zinc supplementation group (group Z) and control group (group C). (**b**) Change in serum zinc levels from admission to 2 weeks postoperatively. Data are presented as mean ± standard deviation. *p < 0.05

Serum zinc levels on admission, the day, one week, and two weeks after surgery did not statistically significantly differ between the groups. However, the change in serum zinc levels from admission to two weeks postoperatively was significantly greater in group Z (Fig. 1, Table 2).

**Table 2:** Changes in serum zinc levels

Time point	Admission	Postoperative day 1	1 week	2 weeks	Change in serum Zinc
Group Z	51.9 ± 11.8	42.6 ± 7.9	70.8 ± 10.5	82.9 ± 21.0	31.0 ± 16.7
Group C	55.7 ± 13.2	42.9 ± 10.8	65.5 ± 12.7	74.0 ± 11.3	20.9 ± 19.1
*P*-value	0.36	0.90	0.12	0.10	0.028

**Fig. 2:**
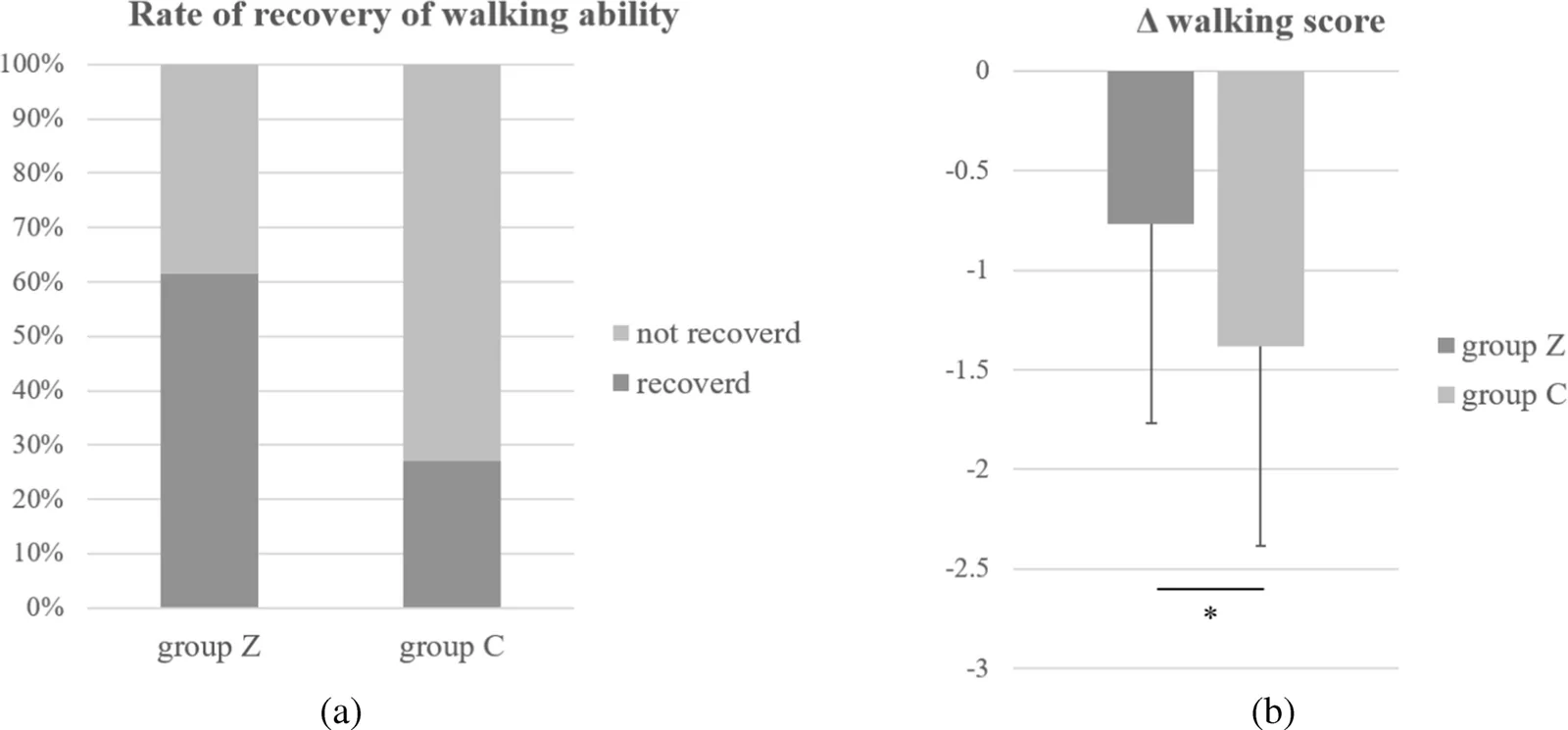
Postoperative functional outcomes. (**a**) Rate of recovery of walking ability at discharge in the zinc supplementation group (group Z) and control group (group C). Recovery was defined as achieving an equivalent or better ambulatory status compared with the pre-fracture level. (**b**) Change in walking score (Δ walking score) between pre-fracture status and discharge. Data are presented as mean ± standard deviation. *p < 0.05

**Fig. 3:**
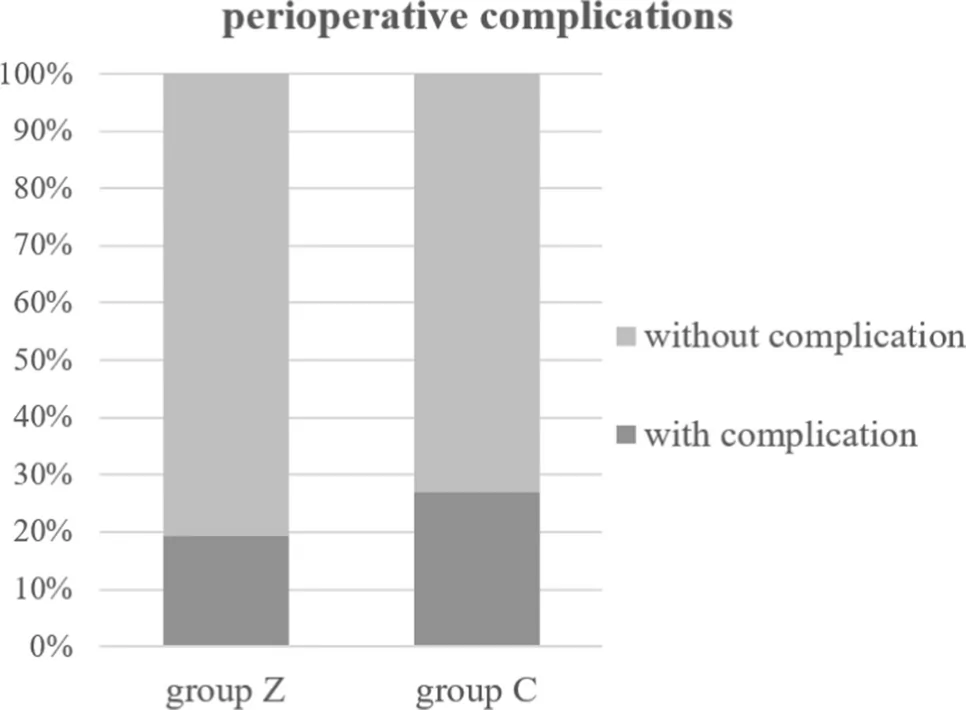
Perioperative complications. Distribution of perioperative complications in the zinc supplementation group (group Z) and control group (group C), presented as 100% stacked bar charts. There was no significant difference between the two groups

The rate of improvement in walking ability was significantly higher in group Z than in group C (61.5% vs. 26.9%, *p* < 0.05). The Δ walking score was also significantly higher in group Z (Fig. 2). There was no statistically significant difference between the two groups in the incidence of perioperative complications (Fig. 3). Serum copper levels were assessed two weeks postoperatively, and no cases of copper deficiency were identified. In addition, no adverse events considered related to zinc supplementation were observed during the study period (Fig. 3).

Multivariable logistic regression analysis was performed to identify independent factors associated with the improvement in walking ability. After adjustment for age, sex, BMI, fracture type, and the presence of postoperative complications, zinc supplementation status was identified as the only independent predictor of improvement in walking ability (OR, 3.74; 95% CI, 1.03–13.70; *p* < 0.05) (Table 3).

**Table 3:** Multivariable logistic regression analysis for factors associated with recovery of walking ability

	Odds ratio	95% CI	*P* value
Age	1.02	0.89–1.16	0.78
Sex (male vs female)	1.18	0.16–8.69	0.87
Body mass index	0.94	0.77–1.15	0.56
Fracture type (trochanteric vs femoral neck)	0.36	0.08–1.61	0.18
postoperative complications	0.39	0.07–2.08	0.27
zinc supplementation status	3.74	1.03–13.70	**0.046**

## Discussion

In this study, we investigated whether zinc supplementation in patients with hip fractures could be associated with improved postoperative recovery of walking ability. The results demonstrated a high prevalence of hypozincaemia in this patient population. Notably, patients who received perioperative zinc supplementation showed a significantly higher rate of walking ability recovery compared with those who did not. Consistently, the Δ walking score was significantly higher in group Z. Furthermore, zinc supplementation remained the only independent predictor of functional recovery using the multivariable logistic regression analysis after adjusting for potential confounders. The extremely high prevalence of hypozincaemia observed in this study may reflect both the poor baseline nutritional status commonly seen in older patients with hip fractures and the acute inflammatory response following trauma. Serum zinc is known to behave as a negative acute-phase reactant and may transiently decrease during systemic inflammation following trauma; therefore, serum zinc levels may not fully reflect total body zinc stores.

Previous studies in non-orthopedic settings have demonstrated the beneficial effects of zinc supplementation on postoperative and functional recovery. A randomized controlled trial showed that perioperative zinc-containing supplementation significantly reduced hospital length of stay in patients who underwent coronary artery bypass graft surgery [6]. Similarly, another randomized controlled trial reported that zinc supplementation improved neurological outcomes in patients with severe traumatic brain injury [7]. These findings suggest that zinc might play an important role in the recovery process following acute injury or surgery. However, despite these promising results, the potential effect of zinc supplementation on functional recovery in patients with hip fractures has not yet been investigated. To the best of our knowledge, this is the first study to demonstrate an association between zinc supplementation and improved postoperative walking ability in this population.

Previous studies have shown that the prevalence of sarcopaenia is significantly higher in patients with low serum zinc levels, suggesting that zinc deficiency might be an independent predictor of sarcopaenia [8]. Moreover, zinc supplementation has been reported to influence the composition of newly synthesized lean tissue and enhance nitrogen metabolism during recovery from malnutrition [9]. Experimental studies have further demonstrated the role of zinc in the skeletal muscle. In animal models of muscle injury, mice fed a low-zinc diet exhibited delayed muscle regeneration, indicating that adequate zinc intake is essential for muscle repair [10]. In addition, zinc reportedly activates the mammalian target of rapamycin signaling pathway, thereby promoting muscle protein synthesis [11]. Taken together, these findings suggest that zinc supplementation might enhance muscle regeneration and protein synthesis, which could contribute to improving walking ability recovery found in the present study.

In patients with hip fractures, improving the nutritional status after hospitalization is often challenging. However, the intervention used in the present study was limited to zinc supplementation initiated at the time of admission, which is simple, safe, and could be readily implemented in clinical practice. These characteristics highlight the potential clinical efficacy of zinc supplementation as a practical perioperative strategy. This study has some limitations. First, the relatively small sample size might limit the generalizability of our findings. Second, this was a single-centre observational study rather than a randomized controlled trial. Although propensity score matching and multivariable analysis were performed, residual confounding cannot be completely excluded because important variables such as cognitive function, frailty, and detailed nutritional status were not available in this study. Additional exploratory multivariable analysis including serum albumin level and time to surgery yielded similar results.

Third, functional outcomes were evaluated only at discharge. Although postoperative rehabilitation was standardized within a single institution and no patients were transferred to rehabilitation hospitals during the acute recovery phase, longer-term outcomes after discharge could not be assessed. Therefore, functional trajectories beyond hospitalization, such as those at six months or one year, remain unclear. Future prospective multicenter studies with long-term follow-up are warranted to confirm the association between zinc supplementation and postoperative functional recovery in patients with hip fractures.

## Conclusion

Patients with hip fractures who received perioperative zinc supplementation showed a significantly higher rate of walking ability recovery. These findings suggest that zinc supplementation might be a simple and effective strategy for improving postoperative functional recovery in this patient population. Further large-scale randomized controlled trials are warranted to confirm these findings and establish the optimal use of zinc supplementation.

## Data Availability

No datasets were generated or analysed during the current study.
